# Supplementary material for: Chronological age, biological age, and individual variation in the stress response in the European starling: a follow-up study
Source: PeerJ. 2018 Oct 23;6:e5842. doi: 10.7717/peerj.5842 (PMC6202956; doi:10.7717/peerj.5842)
Supplement: Supplemental Information 1 — All models contain random effects of natal family. LRT: Likelihood ratio test; * p < 0.05. Age point 1: 127–134 days; age point 2 584–601 days. [file peerj-06-5842-s001.docx]

Table S1. Summaries of statistical models testing for effects of developmental telomere attrition (DTA) on stress response (CORT) variables, in the age point 1 and age point 2 data treated separately. All models contain random effects of natal family. LRT: Likelihood ratio test; * p < 0.05. Age point 1: 127-134 days; age point 2 584-601 days.

| Age point | Outcome | Fixed predictors | B | s.e. (B) | LRT | p-value |
| --- | --- | --- | --- | --- | --- | --- |
| 1 | Baseline CORT | DTA | -0.48 | 0.98 | 0.23 | 0.63 |
|  | Peak CORT | Baseline CORT | 1.10 | 0.93 | 1.36 | 0.24 |
|  |  | DTA | 2.75 | 5.79 | 0.23 | 0.64 |
|  | ΔCORT | CORT 15 mins | -0.57 | 0.12 | 15.75 | <0.001* |
|  |  | DTA | 1.45 | 3.72 | 0.15 | 0.70 |
| 2 | Baseline CORT | DTA | 1.58 | 1.22 | 1.55 | 0.21 |
|  | Peak CORT | Baseline CORT | 0.70 | 0.63 | 1.00 | 0.32 |
|  |  | DTA | -1.99 | 3.98 | 0.22 | 0.64 |
|  | ΔCORT | CORT 15 mins | -0.56 | 0.12 | 15.60 | <0.001* |
|  |  | DTA | 0.91 | 2.62 | 0.11 | 0.74 |
